# 16S rRNA gene based analysis of *Enterobacter sakazakii *strains from different sources and development of a PCR assay for identification

**DOI:** 10.1186/1471-2180-4-43

**Published:** 2004-11-25

**Authors:** Angelika Lehner, Taurai Tasara, Roger Stephan

**Affiliations:** 1 Institute for Food Safety and Hygiene, Vetsuisse Faculty, University of Zurich, CH-8057 Zurich, Switzerland

## Abstract

**Background:**

*E. sakazakii *is considered to be an opportunistic pathogen, implicated in food borne diseases causing meningitis or enteritis especially in neonates and infants. Cultural standard identification procedures for *E. sakazakii *include the observation of yellow pigmentation of colonies and a positive 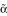
glucosidase activity. Up to now, only one PCR system based on a single available 16S rRNA gene sequence has been published for *E. sakazakii *identification. However, in our hands a preliminary evaluation of this system to a number of target and non-target strains showed significant specificity problems of this system. In this study full-length 16S rRNA genes of thirteen *E. sakazakii *strains from food, environment and human origin as well as the type strain ATCC 51329 were sequenced. Based on this sequence data a new specific PCR system for *E. sakazakii *was developed and evaluated.

**Results:**

By phylogenetic analysis of the new full-length 16S rRNA gene sequence data obtained we could show the presence of a second phylogenetic distinct lineage within the *E. sakazakii *species. The newly developed 16S rRNA gene targeting PCR system allows identification of *E. sakazakii *strains from both lineages. The assay's ability to correctly identify different *E. sakazakii *isolates as well as to differentiate *E. sakazakii *from other closely related *Enterobacteriaceae *species and other microorganisms was shown on 75 target and non-target strains.

**Conclusion:**

By this study we are presenting a specific and reliable PCR identification system, which is able to correctly identify *E. sakazakii *isolates from both phylogenetic distinct lines within the *E. sakazakii *species. The impact of this second newly described phylogenetic line within the *E. sakazakii *species in view of clinical and food safety aspects need further investigation.

## Background

*E. sakazakii *is considered to be an opportunistic pathogen, implicated in food borne diseases causing meningitis or enteritis especially in neonates and infants [1,2]. Mortality rates of 20 – 50% are reported for patients who contract the disease [3]. The survivors often suffer from severe neurological disorders. A recent study on the occurrence of *E. sakazakii *in production environments from food (milk powder, chocolate, cereal, potato, pasta) factories and households isolated the organism with varying frequency from nearly all environments examined, strongly indicating, that this is a widespread organism [4]. Up to now, *E. sakazakii *has been isolated from a wide range of foods, including cheese, meat, vegetables, grains, herbs and spices and UHT milk, but most of the literature concentrates on the presence the organism in dried infant formula milk [5-8]. In 1990, Clark *et al*. [9] were the first to prove a clear epidemiologic correlation between *E. sakazakii *isolated from patients and dried infant formula involved in 2 hospital outbreaks using a combination of typing methods. Although the levels of contamination seemed low, the authors could show, that with an initial concentration of 1 CFU/ml, reconstituted formula stored at room temperature would take approx. 10 hrs to reach 10^7 ^cells per 100 ml and even sooner in formula held on 35 – 37°C. Since then, many case reports have described this epidemiologic correlation [10,11].

Cultural standard identification procedures for *E. sakazakii *include the observation of yellow pigmentation of colonies and the testing for 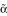
glucosidase activity. Based on this latter biochemical activity commercially selective chromogen media have recently been developed such as the Druggan-Forsythe-Iversen agar, DFI (commercially available under: Chromogenic *Enterobacter sakazakii *agar, Oxoid CM1055, Oxoid, UK) and the Enterobacter Sakazakii Isolation Agar, ESIA (AES Laboratoire, France).

Besides, molecular assays have often proven to be useful as they offer a alternative means to rapidly and specifically identify organisms from a wide variety of sources. Although PCR detection methods are now widely used in identification of microorganisms, only one PCR system, based on a single available full-length 16S rRNA gene sequence of type strain ATCC 29544 [GenBank: AB004746], was published up to now for *E. sakazakii *[12]. However, in our hands a preliminary evaluation of this system on a number of target and non-target strains showed significant specificity problems inherent in this assay. Therefore, the aim of this study was to provide more full-length 16S rRNA sequence data from *E. sakazakii *strains of different origin. Finally, based on this new sequence data a specific PCR system was developed for *E. sakazakii *detection.

## Results and discussion

As a starting point, the full-length 16S rRNA sequences of thirteen *E. sakazakii *strains from various sources (five fruit powder isolates, three human isolates, two production environment isolates, one milk isolate, one baby food isolate, one milk powder isolate) and of *E. sakazakii *type strain ATCC 51329 were determined. This sequence data has been deposited in the GenBank [AY752936 – AY752943, AY803186 – AY803192]. Thereafter, a sequence comparison was performed between the 16S rRNA genes of the fourteen *E. sakazakii *strains and the *E. sakazakii *type strain ATCC 29544 by calculation of a distance matrix using the ARB programme. Thirteen *E. sakazakii *strains used in the study exhibited 99.4 to 100% sequence similarity to the *E. sakazakii *type strain ATCC 29544 [GenBank: AB004746]. Meanwhile similarity between strain ATCC 51329 and the strain ATCC 29544 was significantly lower (97.9%).

**Figure 1:**
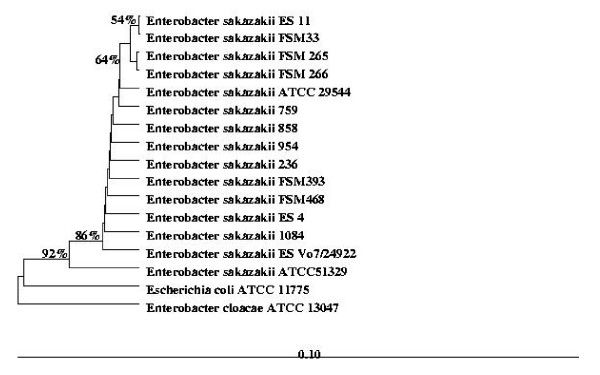
Phylogenetic tree comprising the the 16S rRNA gene sequence data of *E. sakazakii *strains obtained in this study in comparison to *E. sakazakii *type strain ATCC 29544, the *E. cloacae *type strain ATCC 13047 and the *E. coli *type strain ATCC 11775. The scale bar represents ten nucleotide substitutions per 100 nucleotides.

Phylogenetic affiliation of the newly obtained sequences to tree_jul04_1450 comprising >28.000 almost full-length (>1449 nucleotides) 16S rRNA gene sequences revealed the presence of two phylogenetically distinct lineages within the *E. sakazakii *species. In figure 1 a subtree based on the data mentioned above is shown. From this analyses it can be observed, that thirteen of the fourteen newly sequenced strains cluster together with the *E. sakazakii *type strain ATCC 29544, whereas the *E. sakazakii *strain ATCC 51329 forms a second branch within this group. The subtree was calculated using the TREEPUZZLE tool within the ARB package exhibiting a "consensus tree" from different calculation methods. The value on each branch is the percent occurrence of the branching order in 500 bootstrapped trees. The 92% bootstrap value for the *E. sakazakii *ATCC 51329 branch strongly supports the theory about the second lineage.

Further qualitative inspection of the sequences revealed that, besides of the presence of a number of single base substitutions, at least four "hot spots" of polymorphisms can be observed along the *E. sakazakii *16S rRNA gene (*E. coli *positions 187 to 193; 455 to 477, 590 to 600 and 1132 to 1141). Within these regions substitutions ranging from two to seven bases are present in all strains investigated and are most pronounced in *E. sakazakkii *strain ATCC 51329, thus providing the bases for the formation of the second phylogenetic branch. In table 1, the sequence polymorphisms in the above mentioned regions are shown for all *E. sakazakii *strains investigated in the study in relation to the *E. sakazakii *typestrain ATCC 29544, *E. cloacae *typestrain ATCC13047 and *E. coli *type strain ATCC 11775. Recently, a number of *E. sakazakii *partial sequences were deposited into the public database [13]. Addition of these partial sequences (mostly the first 500 bp of the 16S RNA gene) to the phylogenetic tree further confirms the presence of the two lineages within the *E. sakazakii *species (data not shown). However, for a reliable pylogenetic analyses determination of at least 1000 nucleotides is recommended [14]. The impact of this second phylogenetic line within the *E. sakazakii *species in view of clinical and food safety aspects needs further evaluation.

In a second step, based on our new sequence information, we designed a specific primer pair for the amplification of the 16S rRNA gene of strains within the two phylogenetic lineages of *E. sakazakii*. They were first evaluated using PROBE-MATCH tool of the ARB software. Additionally, BLAST searches were performed against the non-redundant database (nr) of EMBL/GENBANK. Both primers seemed to be specific. Amplification conditions in the assay were optimized by taking an annealing temperature of 60°C for 1 min.

**Figure 2:**
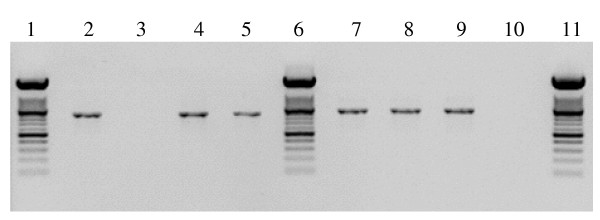
Agarose gel analyses of selected target and non-target strains after amplification of the DNA using the PCR system established by Keyser et al. [10] and the system developed in this study. Lane 1, 6, 10: MWM (Roche XIV), lane 2: *E. sakazakii *ATCC 29004, 3: *E. sakazakii *ATCC 51329, 4: *E. sakazakii *fruit powder isolate, 5: *E. cloacae*, wild strain amplified with the Keyser PCR system; lane 7 – 10 same strains amplified with the system developed in this study.

We then sought to evaluate the utility of the PCR assay next. In particular the assay's ability to correctly identify different *E. sakazakii *strains as well as to differentiate *E. sakazakii *from other closely related *Enterobacteriacae *was assessed. This was done by applying the assay to purified genomic DNA template from isolates of 45 known *E. sakazakii *strains form different origin and 28 non *E. sakazakii *strains. A gel-based example of the screen is shown in figure 2, where *E. sakazakii *isolates and non-target microorganisms were analyzed. The rest of the results from the screen are summarized in table 2. The new PCR assay was able to correctly confirm all the *E. sakazakii *isolates tested. There were no amplification products observed when the non *E. sakazakii *strains controls were similarly analyzed.

For comparison the various strains were also similarly analyzed using the PCR system described by Keyser et al. [12]. The results are summarized in table 2 and confirm our preliminary evaluation data where we found significant specificity problems with this system. A gel-based example of the screen is shown in figure 2, where *E. sakazakii *isolates and non-target microorganisms were analyzed. First the system was not able to detect all the *E. sakazakii *strains. By using the PROBE_MATCH tool included in the ARB software it can be retrieved, that one of the primers (Esak3) used in the Keyser system exhibits four mismatches at the 3 prime end to the 16S rRNA gene sequence of *E. sakazakii *strain ATCC 51329, thus providing a possible explanation for the negative amplification result with this strain. Moreover, this system also gives positive results with some non-target organisms (e.g. *E. cloacae*, *S. liquefaciens*, *S. fucaria*, *S*. Enteritidis).

**Figure 3:**
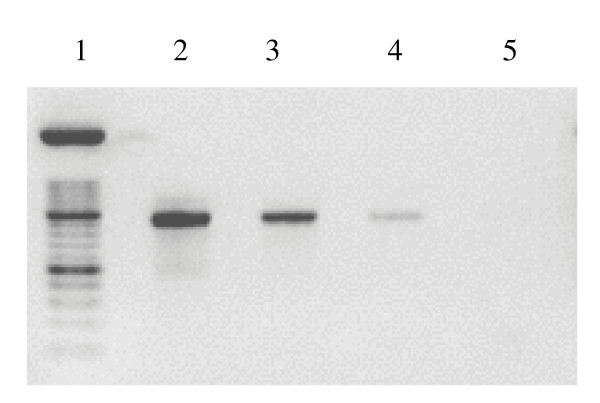
Defining the detection limit of the PCR system. Decreasing amounts of purified *E. sakazakii *strain ATCC 51329 genomic DNA target (100 ng – 1 pg) were amplified by PCR. Lane 1, DNA 100 bp marker; In lanes 2 to 5, 1 ng, 100 pg, 10 pg, 1 pg of *E. sakazakii *DNA was used per reaction.

To determine the detection limit of the newly developed assay, PCR was performed on decreasing amounts of purified DNA template from representatives of the two *E. sakazakii *lineages (strain ATCC 51329 and strain ATCC 29544). In both strains a detection limit of 10 pg was determined (figure 3). Furthermore the influence of non-specific DNA background on the assay's detection limit and efficiency was also investigated. The target DNA template concentration was held constant at 10 pg while increasing amount of non-target DNA, purified templates from *B. cereus *ATCC 10876, *L. acidophilus *ATCC 13651, *S. aureus *ATCC 25923 and *E. faecium *DSM 2918 were used. There were no significant influences on assay's performance observed in the presence of up to 200 ng non-target DNA in the reaction mixture (data not shown).

## Conclusions

By phylogenetic analysis of the new provided full-length 16S rRNA sequence data we could show the presence of a second phylogenetic distinct lineage within the *E. sakazakii *species. Based on this new sequence data we have developed a 16S rRNA targeting PCR assay, which allows identification of *E. sakazakii *strains from both lineages. The assay's ability to correctly identify different *E. sakazakii *isolates as well as to differentiate *E. sakazakii *from other closely related *Enterobacteriaceae *species and other non-target microorganisms was shown. This PCR system provides a valuable tool for rapid identification of this food borne pathogen.

## Methods

### Bacterial strains

Overall, 47 *E. sakazakii *strains, with isolates from human, food and environmental origin, as well as 28 non *E. sakazakii *strains from selected species were included in this study (Table 2). In a first step, the 16S rRNA genes of fourteen *E. sakazakii *strains (*E. sakazakii *ATCC 51329, *E. sakazakii *1084 fruit powder isolate, *E. sakazakii *954 fruit powder isolate, *E. sakazakii *858 fruit powder isolate, *E. sakazakii *759 fruit powder isolate, *E. sakazakii *236 fruit powder isolate, *E. sakazakii *FSM393 baby food isolate, *E. sakazakii *FSM33 milk isolate, *E. sakazakii *265 milk powder isolate, *E. sakazakii *FSM468 production environment isolate, *E. sakazakii *266 production environment isolate, *E. sakazakii *ES4 human isolate, *E. sakazakii *ES11 human isolate, *E. sakazakii *ES Vo7/24922 human isolate) were sequenced. Afterwards, all strains were used for validation of the specificity of the new developed PCR identification system.

The strains were grown on blood agar plates under appropriate conditions. DNA was extracted from the grown colonies using the DNeasy^R ^Tissue Kit (Qiagen AG, Switzerland) in accordance with the suppliers' protocol.

### 16S rRNA amplification and direct sequencing

For 16S rRNA gene amplification, reaction mixtures (total volume 50 μl) containing primers 616V (5' AGA GTT TGA TYM TGG CTC 3') and 630R (5' CAK AAA GGA GGT GAT CC 3') [15] at 10 pmol each were prepared by using the Expand High Fidelity PCR system (Roche, Rotkreuz, Switzerland): 10 × Expand High Fidelity buffer (without MgCl_2_), 2.5 mM MgCl_2_, 200 μM dNTPs each and 3 U Expand High Fidelity enzyme mix.

The amplification was performed in a T3 thermocycler (Biometra, Germany). The PCR conditions were: 2 min at 95°C, 10× (94°C, 15 s; 52°C, 30 s; 72°C, 90 s) followed by 15× (94°C, 15 s; 52°C, 30 s; 72°C, 129 s). Cycling was completed by a final elongation step at 72°C for 7 min. After PCR the reaction products were separated on a 1.5% agarose gel, stained with ethidium bromide and visualized under UV light. In cases where sequencing was desired, the correct size (approx 1500 bp) products were excised from the gel and purified using the MinElute™ gel extraction kit (Qiagen, Switzerland). The products were thereafter sequenced. Sequencing reactions were performed using a modified Sanger method and the Big-Dye chemistry from Applied Biosystems on an ABI 3730 capillary DNA Analyzer (Applied Biosystems, USA) employing the same primer pair as for amplification of the 16S rRNA gene (616V/630R) and additional internal primers for "walking reactions". Sequencing was performed by Microsynth (Balgach, Switzerland).

### Phylogenetic analysis, tree construction and design of specific primer

16S rRNA gene sequences of fourteen newly sequenced strains were added to an alignment of about 28'000 almost full length small subunit rRNA sequences by using the alignment tool of ARB program package [16]. Alignments were refined by visual inspection. Phylogenetic analyses were performed by using distance matrix and the TREEPUZZLE tool of the ARB program. Primer design was accomplished by applying the PROBE DESIGN tool included in the software package ARB on special data structures (PT-Servers) derived from the ssu-rRNA database "ssu_jan04.arb".

The following specific *E. sakazakii *S16 rRNA gene targeting primers Esakf (5' GCT YTG CTG ACG AGT GGC GG 3') and Esakr (5' ATC TCT GCA GGA TTC TCT GG 3') were designed and synthesized (Mirosynth, Balgach, Switzerland). This primer pair binds to conserved regions (*E. coli *position 88 – 107 (Esakf) and 1017 – 998 (Esakr)) in the S16 rRNA gene sequences giving an amplicon of 929 bp.

### PCR reaction conditions

For amplification, reaction mixtures (total volume 50 μl) containing primer Esakf and Esakr at a concentration of 10 pM were prepared by using 10 × *Taq *reaction mixture, 2 U *Taq *polymerase (Promega, Madison, WI),) and 200 μM dNTPs each. Thermal cycling was carried out by using an initial denaturation step of 94°C for 2 min, followed by 29 cycles of denaturation at 94°C for 30 sec. annealing temperature for 1 min and elongation at 72°C for 1 min 30 sec. Cycling was completed by a final elongation step at 72°C for 5 min. Amplification conditions were optimized by gradually increasing the annealing temperature in the assay from 52°c to 64°C. The reaction products were resolved on a 1.5% agarose gel followed by ethidium bromide staining and examination under UV light.

### Accession number of 16S rRNA sequences

*E. sakazakii *ATCC 51329 [GenBank: AY752937], *E. sakazakii *1084 fruit powder isolate [GenBank: AY803192], *E. sakazakii *954 fruit powder isolate [GenBank: AY752938], *E. sakazakii *858 fruit powder isolate [GenBank: AY752936], *E. sakazakii *759 fruit powder isolate [GenBank: AY752939], *E. sakazakii *236 fruit powder isolate [GenBank: AY752943], *E. sakazakii *FSM393 baby food isolate [GenBank: AY752941], *E. sakazakii *FSM33 milk isolate [GenBank: AY752940], *E. sakazakii *265 milk powder isolate [GenBank: AY803191], *E. sakazakii *FSM468 production environment isolate [GenBank: AY752942], *E. sakazakii *266 production environment isolate [GenBank: AY803190], *E. sakazakii *ES4 human isolate [GenBank: AY803186], *E. sakazakii *ES11 human isolate [GenBank: AY803187], *E. sakazakii *ES Vo7/24922 human isolate [GenBank: AY803189].

## Authors' contributions

AL and TT carried out the experimental part of the study. RS carried out the conception of the study. All authors participated in production and approval of the manuscript.

## Figures and Tables

**Table 1:** Polymorphism "hot spots" along the 16S rRNA gene of the *E. sakazakii *strains analyzed in the study in correspondence to *E. sakazakii *type strain ATCC 29544, *E. coli *type strain ATCC 11775 and *E. cloacae *type strain ATCC13047

			*E. sakazakii *16S rRNA gene sequence polymorphism positions^1^			
			
strain	Sequence accession no.	source	I (187–193)	II (455–477)	III (590–600)	IV (1132–1141)
*E. coli *ATCC 11775	X80725		GCAAGCA	GAGTAAAGTTAATACCTTTGCTC	TTGTTAAGTCA	CGGTCCG-GCC
*E. cloacae *ATCC 13047	AJ251469		GCAAGAC	TGTTGTGGTTAATAACCGCAGCA	CTGTCAAGTCG	CGGTCCG-GCC
*E. sakazakii *ATCC 29544	AB004647	human	TACGGAC	TGTTGTGGTTAATAACCGCAGCA	TGATTAAGTCA	CGGTTCG-GCC
*E. sakazakii *ES 11	AY803187	human	TWCGGAC	YGYTGTGGTTAATAACCACAGCA	CTGTTAAGTCA	CGGTTCG-GCC
*E. sakazakii *ES 4	AY803186	human	TWCGGAC	TGYTGTGGTTAATAACCACAGCA	CTGTTAAGTCA	CGGTCCG-GCC
*E. sakazakii *FSM 266	AY803190	environment	TACGGAC	CGTTGTGGTTAATAACCGCAGCG	CTGTTAAGTCA	CGGTTCG-GCC
*E. sakazkaii *1084	AY803192	fruit powder	TACGGAC	CGTTGTGGTTAATAACCACAGCG	YKRTTAAGTCA	CGGTTCG-GCC
*E. sakazakii *ES Vo7/24922	AY803189	human	TACGGAC	TGTTGTGGTTATTAACCRCAGCA	YKRTTAAGTCA	CGGTTCG-GCC
*E. sakazakii *FSM 265	AY803191	milk powder	TTCGGAC	CGTTGTGGTTAATAACCGCAGCG	YKRTTAAGTCA	CGGTTCG-GCC
*E. sakazakii *ATCC 51329	AY752937		GCAAGAC	GGTTAAGGTTAATAACCTTGGCC	CTGTCAAGTCG	CACATCATGGT
*E. sakazakii *858	AY752936	fruit powder	TACGGAC	TGTTGTGGTTAATAACCGCAGCA	TGATTAAGTCA	CGGTCCG-GCC
*E. sakazakii *759	AY752939	fruit powder	TACGGAC	TGTTGTGGTTAATAACCACAGCG	CTGTTAAGTCA	CGGTYCG-GCC
*E. sakazakii *954	AY752938	fruit powder	TWCGGAC	YGYTGTGGTTAATAACCACAGCR	YTGTTAAGTCA	CGGTYCG-GCC
*E. sakazakii *236	AY752943	fruit powder	TWCGGAC	YGYTGTGGTTAATAACCACAGCR	YKGTTAAGTCA	CGGTYCG-GCC
*E. sakazakii *FSM 468	AY752942	environment	TACGGAC	YGYTGTGGTTAATAACCACAGCA	YTRTTAAGTCA	CGGTTCG-GCC
*E. sakazakii *FSM 393	AY752941	baby food	TACGGAC	CGTTGTGGTTAATAACCGCAGCG	TKRTTAAGTCA	CGGTCCG-GCC
*E. sakazakii *FSM 33	AY752940	milk	TTCGGAC	TGTTGTGGTTAATAACCACAGCA	CTGTTAAGTCA	CGGTTCG-GCC

^1 ^*E. coli *positions according to Brosius et al. [17]

**Table 2:** Bacterial strains used in the study and results of the PCR identification system by Keyser et al. [12] in comparison to the system established in this study

No of strains	species	origin	PCR result (primers from this study)	PCR results (Keyser primer set)
1	*E. sakazakii*	ATCC 29004	+	+
1	*E. sakazakii*	ATCC 51329	+	**negativ**
1	*E. sakazakii*	ATCC 29544	+	+
10	*E. sakazakii*	fruit powder	+	+
1	*E. sakazakii*	milk powder	+	+
2	*E. sakazakii*	baby food	+	+
1	*E. sakazakii*	milk	+	+
16	*E. sakazakii*	production environment	+	+
14	*E. sakazakii*	human	+	+
1	*E. cloacae*	LMG 2783	-	-
1	*E. cloacae*	LMG 3008	-	-
1	*E. cloacae*	food	-	-
1	*E. cloacae*	food	-	**positive**
1	*E. cloacae*	clinical	-	-
1	*P. dissolvens*	LMG 2683	-	-
1	*P. agglomerans*	LMG 1286	-	-
3	*P. agglomerans*	food, clinical, cosmetics	-	-
1	*E. hermanii*	wild strain	-	-
1	*E. coli*	ATCC 25922	-	-
1	*E. coli*	wild strain	-	-
1	*S. liquefaciens*	cosmetics	-	**positive**
1	*S. fucaria*	production environment	-	**positive**
1	*K. oxytoca*	cheese	-	-
1	*K. pneumoniae*	wild strain	-	-
1	*P. mirabilis*	DSM 788	-	-
1	*S. sonnei*	ATCC 29930	-	-
1	*S*. Enteritidis	wild strain	-	**positive**
1	*P. aeruginosa*	ATCC 15442	-	-
1	*L. acidophilus*	ATCC 13651	-	-
1	*S. aureus*	ATCC 25923	-	-
1	*S. agalactiae*	ATCC 33019	-	-
1	*B. cereus*	ATCC 10876	-	-
1	*E. faecium*	DSM 2918	-	-
1	*L. monocytogenes*	wild strain	-	-
1	*M. luteus*	ATCC 9341	-	-
